# Accidental Death from Morphine

**Published:** 1898-02

**Authors:** 


ACCIDENTAL DEATH FROM MORPHINE.—The subject was a horse, two and one-half years, and had undergone an operation for abdominal cryptorchidism. After getting up, a hernia appeared which was reduced. In order to keep the animal quiet, and while he was still suffering from the effects of the chloroform, Ries injected subcutaneously fifteen grains of morphine.

Three-quarters of an hour afterward the animal became uneasy, perspired freely, and had a distressed eye, tremblings, and convulsions. Two hours later the convulsions became very violent and terminated in death after a short agony.

The author thinks that the dose of morphine was too large, and that we cannot be guided by the action of this drug in man.—*Ann. de Méd. Vét.*

